# The influence of regularly changing enrichment on the cognitive judgement bias of laboratory rats

**DOI:** 10.1038/s41598-025-22088-x

**Published:** 2025-10-08

**Authors:** Elena Groneberg, Linn N. Hoppe, S. Helene Richter, Sylvia Kaiser

**Affiliations:** 1 Department of Behavioural Biology, University of Münster, Badestrasse 13, 48149 Münster, Germany; 2 DFG Research Training Group EvoPAD, University of Münster, Münster, Germany

**Keywords:** Enrichment, Refinement, Welfare, Cognitive judgement bias, Laboratory rat, Ecology, Ecology, Neuroscience, Psychology, Psychology, Zoology

## Abstract

The provision of environmental enrichment is a frequently used refinement method to improve welfare of laboratory animals. Compared to barren housing conditions, enrichment could decrease signs of impaired welfare, such as stress hormone concentrations and stereotypies. Moreover, the provision of enrichment was found to positively affect animals’ emotional states, reflected by more optimistic judgements in cognitive bias paradigms. To investigate the impact of environmental enrichment beyond the classical comparison between barren and enriched housing, the aim of this study was to examine the influence of two different enrichment regimes on the cognitive judgement bias of laboratory rats (*Rattus norvegicus* f. *domestica)*. Twenty-one individuals were housed either under consistent enrichment or regular enrichment change for several months. Afterwards, they participated in a cognitive judgement bias test, which is based on ambiguous cue interpretations and enables a classification of rats as being optimistic or pessimistic. Interestingly, we found that rats from the enrichment-change group responded more optimistically in an ambiguous situation than rats from the consistent-enrichment group. It seems that the regular enrichment change could induce a more positive emotional state in our rats and, therefore, represents a simple but effective approach to improve welfare under laboratory conditions.

**Supplementary Information:**

The online version contains supplementary material available at 10.1038/s41598-025-22088-x.

## Introduction

As an integral part of the 3R principle, refinement aims to alleviate any suffering and distress during experimental procedures and to improve the living conditions of laboratory animals^1^. A widely used refinement strategy is the provision of environmental enrichment^2,3^. Enrichment, thereby, summarises any modification to the environment that enhances species-specific needs and behaviours (e.g. provision of nesting material or shelter) by increasing the complexity of the housing environment^4^. The positive effect of enrichment on laboratory animals was proven in various studies, indicated for example by a reduction of anxiety-like behaviours and stereotypies in mice and rats or a reduction of hormonal stress reactions^5–9^. However, it should be noted that the relationship between stress hormones and enrichment is complex, as glucocorticoids have catabolic effects: for example, restrictive housing conditions can lead to a decrease of stress hormone release as a consequence of boredom, while an enriched housing system can lead to an increase in stress hormone release because of a higher activity and thus higher energy requirements^10^.

According to Broom (1991), the term animal welfare refers to an animal’s state in relation to its environment. Coping with the environment is essential and difficulties or failure to cope would result in poor animal welfare. Thereby, welfare should be regarded as a continuum, varying from good welfare on one end to poor welfare on the other^11^. In addition to physiological indicators of animal welfare, psychological influences should also be taken into account. Whereas most enrichment studies used mainly physiological and behavioural indicators of animal welfare, researchers also place more and more emphasis on the inclusion of animals’ emotional states, when investigating animal welfare^12–14^. It is known that emotional states are able to influence an animal’s cognition, which opens up the possibility to assess so called cognitive biases as indicators of emotional states^14^. It is assumed, for example, that animals in a negative emotional state will interpret an ambiguous cue as negative (pessimistic response), whereas a positive emotional state is more likely to lead to a positive interpretation (optimistic response)^15^. A frequently used method to test such interpretations of ambiguous cues, is the cognitive judgement bias test (CJBT)^14,16^. So far,  CJB tests have been applied successfully in various animal species^17–21^ and were also used to investigate the impact of enriched housing conditions across species. Rats, for example, were more optimistic after being transferred to enriched cages^22,23^ and starlings housed in enriched cages showed increased optimism compared to individuals living in standard housing conditions^24^. Another study revealed that pigs, which were first housed in barren conditions, responded more optimistically to an ambiguous cue after being transferred to an enriched pen^25^. Moreover, the latency to respond to the ambiguous cue increased after the pigs were returned to barren housing, which indicates a negative emotional state in response to enrichment loss^25^. However, there are also contrasting results. A recent study incorporated different housing conditions in zebrafish but the results of the CJBT did not seem to be strongly affected by the provision of enrichment^26^. Two other studies found similar results. Grizzly bears, for example, showed no change in optimism after having access to different enrichment items^27^, and pigs from enriched and barren housing condition did not differ in their optimism levels^28^.

Strikingly, most studies investigating the effect of enriched housing conditions compared very reduced barren housing with the provision of enrichment. However, it might be interesting to go a step further and extend the investigation to different enrichment regimes. Under natural conditions, animals are usually exposed to frequent changes in their environment^29,30^. Therefore, a less rigid composition of cages might be an interesting starting point. Specifically, exploring different forms of enrichment presentation, could be particularly promising to mimic natural environments more closely. Against this background, some studies investigated whether the environmental complexity or the novelty of enrichment items contribute to the improvement of animal welfare. Abou-Ismail and Mendl^31^, for example, either presented one novel item (but multiple copies of this item) per week to their rats or offered a combination of different enrichment items that remained unchanged over several weeks. Interestingly, not the novelty of the enrichment item, but the consistent provision of a combination of different items could positively affect welfare-related parameters^31^. Likewise, another study on mice showed that access to additional enrichment was generally preferred, but no difference in preference between consistent enrichment or frequently changing novel enrichment combinations was found^32^.

The current study expands upon this approach by investigating the effect of two different enrichment regimes, consistent enrichment and regular enrichment change, on the judgement bias of laboratory rats. In contrast to other CJBT studies focusing on the provision of enrichment, all individuals had excess to environmental enrichment before and during the experimental phase. However, only half of the individuals experienced a weekly change of enrichment items. A tactile CJBT was used to determine the optimism level of the rats as a measure of their emotional states in response to the two enrichment regimes. We hypothesised that the two treatment groups (consistent-enrichment and enrichment-change) differ in the number of optimistic responses in the ambiguous test conditions.

## Materials and methods

### Animals and housing conditions

**Fig. 1:**
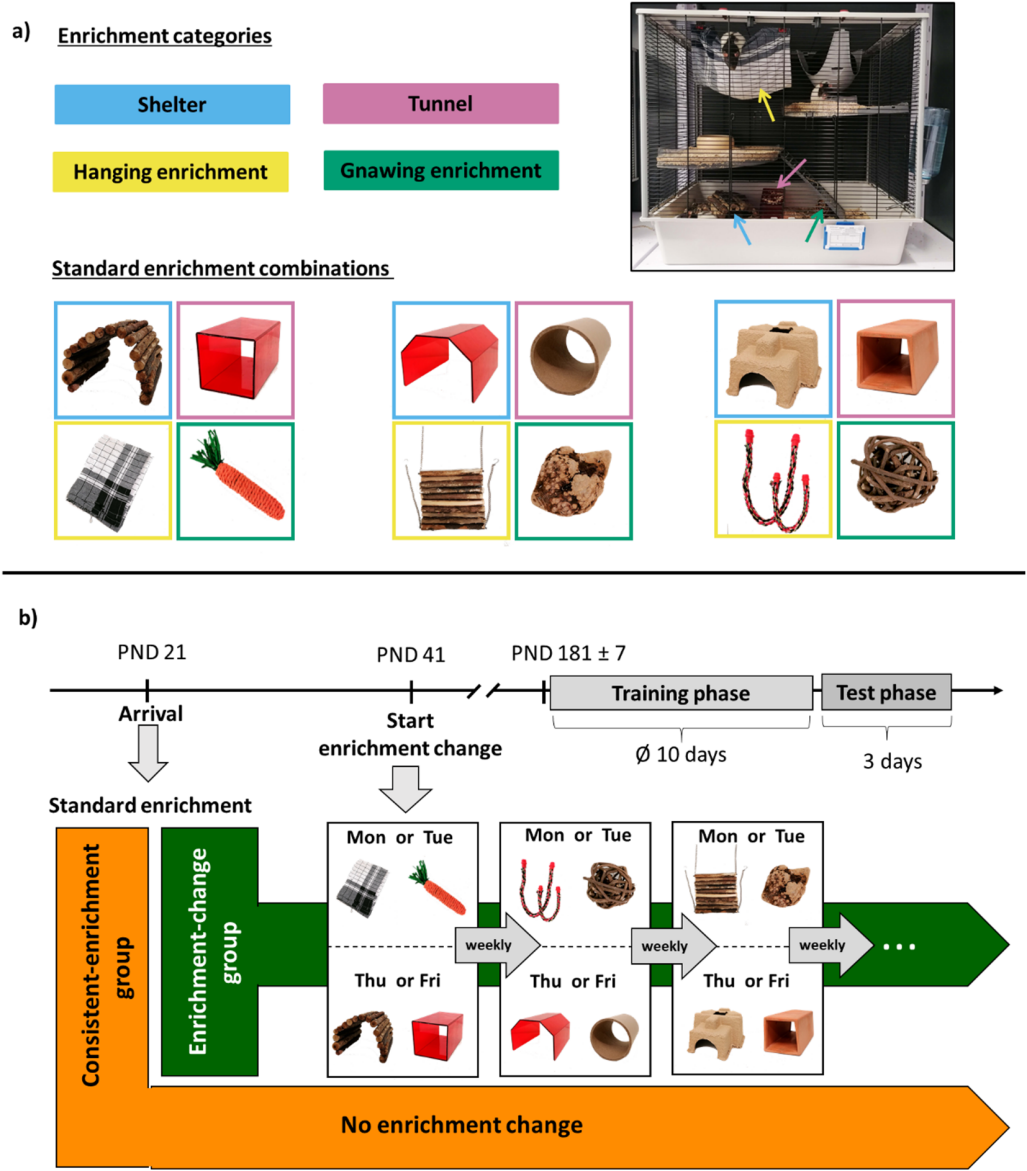
Timeline, enrichment and treatment groups. (**a**) Enrichment items were classified in four different categories: shelter (blue), tunnel (pink), hanging enrichment (yellow) and gnawing enrichment (green). The positions within the cage are indicated by coloured arrows. On the day of arrival rats were assigned to one of three different enrichment combinations. These enrichment combinations remained the same for the first three weeks. (**b**) Rats arrived at PND 21 and were assigned to a treatment group (consistent-enrichment or enrichment-change). On PND 41, the weekly enrichment change started but only for the enrichment-change group. The consistent-enrichment group maintained their enrichment until the end of the test phase. Enrichment items were exchanged twice a week. The combination of enrichment items had to be different from the combination the week before and the week thereafter. The training phase for the CJBT started on PND 181 ± 7. By then the rats had experienced regular enrichment change for approx. 5 months. Training lasted on average 10 days and was directly followed by 3 days of cognitive judgment bias testing.

For this study, 21 female Lister Hooded rats were used. All rats were purchased from a professional breeder (Charles River Laboratories Research Models and Services, Germany GmbH, Sulzfeld, Germany) and participated in another behavioural study before^33^. The rats were 3 weeks old (postnatal day (PND) 21), when they arrived at the institute. Individual fur colouration was used for identification; therefore, no additional markings were needed. All animals were kept under a 12 h dark/light rhythm with lights off at 9 a.m. Ambient temperature and humidity were maintained at about 22 °C and 55%. Rats were housed in groups of three or four individuals in large wire cages (Furat, Ferplast, Italy; 48 cm x 78 cm x 70 cm) leading to a total number of 6 cages. Each cage had two additional levels and wood shavings were used as bedding material (AllSpan German Horse Super, AllSpan German Horse Vertrieb GmbH und Co.KG, Germany). All cages were enriched with one hanging house (Sputnik) (SAVIC, Belgium; 29 cm x 26 cm x19 cm) at the top of the cage, shredded cardboard (Sizzle nest, ZOONLAB GmbH Animal Husbandry Experts, Germany) as nesting material and a wooden ball and cube (ZOONLAB GmbH Animal Husbandry Experts, Germany) for gnawing. Additionally, all cages contained four more enrichment items, which could be divided in four categories: shelters, tunnels, items for gnawing and hanging enrichment items for climbing or resting (see “Enrichment change”, Fig. 1a). Cages and enrichment items were cleaned once a week. Food (Altromin 1324, Altromin GmbH, Germany) and water were provided *ad libitum*. During the training and test phase of the CJBT, rats were mildly food restricted to increase motivation to participate. Individuals were maintained at 90–95% of their *ad libitum* body weight. Therefore, rats were weighed every morning before training or testing to keep track of their weight and to determine the appropriate amount of food per cage. Since rats were not tested or weighed over the weekend, the amount of food for the weekend was based on the bodyweights measured on Friday. Food restriction started 4 days prior to the training phase and continued until the last rat of the cage finished the test phase. The weighed amount of food was provided once a day in the afternoon after the last individuals were finished with either training or testing.

## Experimental design

To investigate the effect of different enrichment regimes on the cognitive judgment bias of rats, individuals were divided in two groups: consistent-enrichment group and enrichment-change group. Each cage of both groups was assigned to a standard enrichment combination (see Fig. 1 and “Enrichment change”) on the day of arrival. For the consistent-enrichment group, these enrichment items remained the same throughout the whole experimental phase. For the enrichment-change group, the standard enrichment was only maintained for 3 weeks. Afterwards, enrichment items were changed twice a week due to the experimental design of the previous study^33^ (see ”Enrichment change”). Training for the CJBT (including habituation to the test apparatus) started when the rats were about 6.5 months old (PND 184 ± 7). All rats had participated in other behavioural tests before, which focused on lateralised behaviours (for review see^33^, but had a test-free interval of at least 9 weeks between previous tests and onset of CJBT training. Rats were trained daily, except weekends, until they reached a learning criterion of 80% correct trials (for details see “Training phase” and Table 1). Afterwards, the cognitive judgment bias test was conducted on three consecutive days (for details see “Test phase”).

## Enrichment change

In total, 12 different enrichment items were used in this study to create varying enrichment combinations. The items were classified in four categories: shelters, tunnels, hanging enrichment and gnawing enrichment. Items within each category differed in material and/ or shape (see Fig. 1a; for more detailed description of the enrichment items see “Supplementary Material”). Before arrival of the animals, three standard enrichment combinations were defined, which differed from each other and contained one item of each enrichment category (Fig. 1a). Therefore, each standard enrichment combination was represented once in both treatment groups. From PND 41 onwards, enrichment was changed twice a week for the enrichment-change group. At the beginning of the week (Mondays or Tuesdays), we changed the hanging and gnawing enrichment items, and at the end of the week (Thursdays or Fridays) shelter and tunnel were exchanged (Fig. 1b). Since the previously conducted study investigated the influence of environmental novelty on lateralised behaviour in rats^33^, the enrichment change followed specific rules to maintain a high level of novelty. All items had to be different from the items used the week before and the week after. Moreover, the presentation of similar objects together within one combination, like red shelter and red tunnel or cardboard shelter and cardboard tunnel, was avoided to keep the combinations as interesting and varied as possible. In total, individuals of the enrichment-change group had experienced weekly enrichment change for 19–21 weeks before the CJBT training started. We were not able to present completely novel enrichment combinations every week, since enrichment combinations were inevitably repeated due to the long timespan of enrichment provision. Nevertheless, we made sure that items did not directly repeat from one week to the next until the end of the test phase.

## Cognitive judgement bias test (CJBT)

To determine whether rats of both groups interpret an ambiguous stimulus in an optimistic or pessimistic way, we used a modified version of the cognitive judgement bias test developed by Brydges and Hall^34^. In general, a CJBT is based on the interpretation of an ambiguous cue in reference to previously conditioned positive and negative cues. In our case, we used tactile cues, namely differently grained sandpapers. During a training phase, rats learned to associate coarse sandpaper (type 60) and fine sandpaper (type 1200) with either a big reward (positive condition) or a small reward (negative condition). During the test phase, rats were introduced to three unknown and ambiguous sandpapers (near-coarse, middle, near-fine). Based on their response, we could deduce whether they anticipated a small or a big reward, hence whether they responded optimistically or pessimistically in an ambiguous situation.

## Test setup and test paradigm

**Fig. 2:**
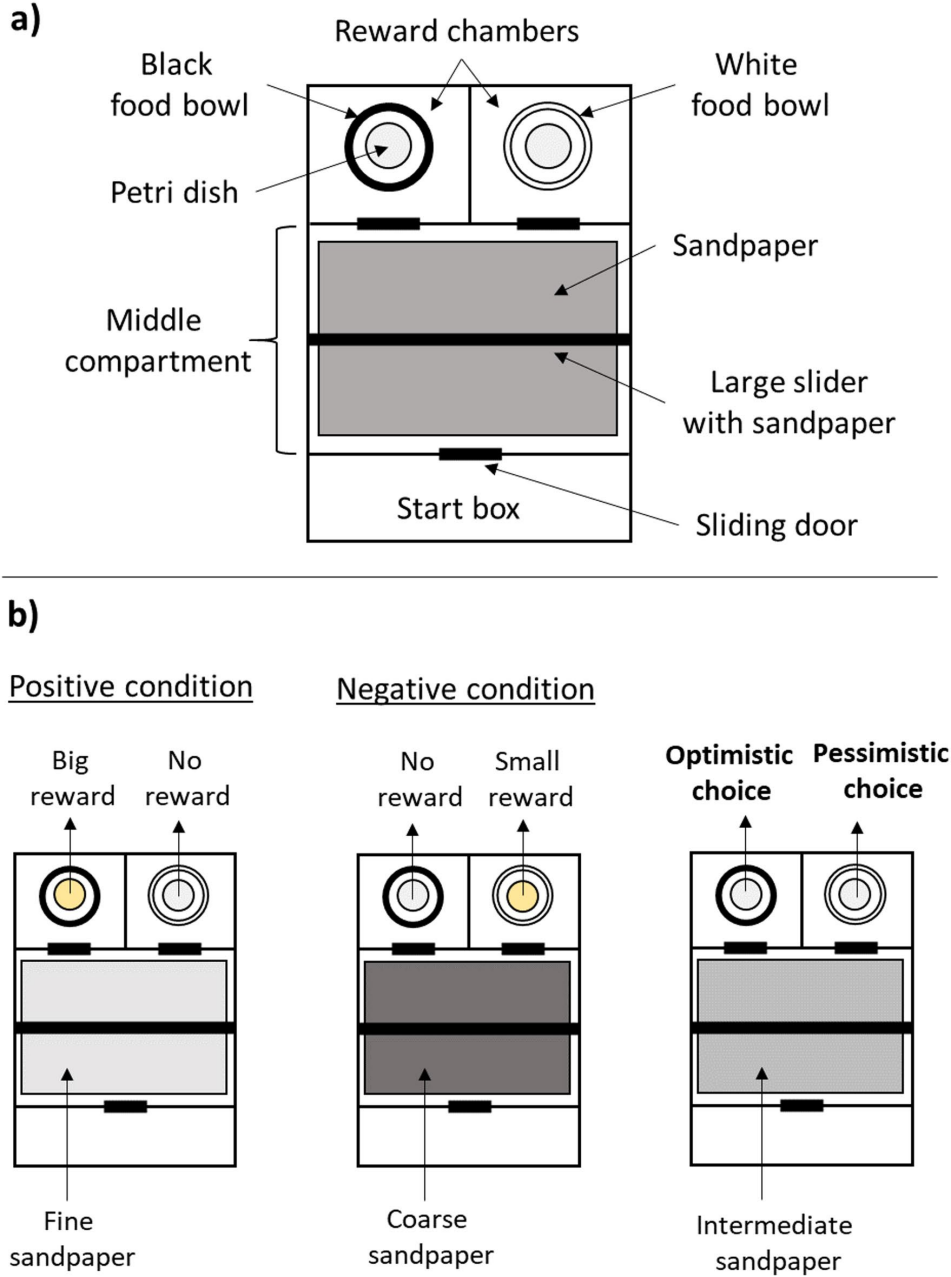
Cognitive judgement bias test (CJBT). (**a**) Schematic illustration of the CJBT setup. The apparatus is divided in start box, middle compartment and two reward chambers. Sandpaper is used as a tactile cue on the floor of the middle compartment and on the large slider. Food rewards are presented on top of the petri dishes in the reward chambers. All compartments are connected by small sliding doors. (**b**) Schematic illustration of the CJBT paradigm. Fine sandpaper is used for the positive condition and a big reward is presented in the left reward chamber. For the negative condition, coarse sandpaper and a small reward in the right reward chamber are used. If a rat enters the left reward chamber when intermediate sandpaper is presented, its choice would be interpreted as optimistic (expectation of a big reward). A decision for the right reward chamber would represent a pessimistic choice (expectation of a small reward).

The test apparatus consisted of a type IV Makrolon cage without a cage lid. To prevent the rats from jumping out of the apparatus, a frame made of acrylic plastic was added to increase the height of the walls to 35 cm. The apparatus was divided in four compartments by inserting non-transparent plastic walls. The start chamber (33 cm x 13 cm) was the only compartment with a transparent lid made of Plexiglas. Through a squared opening (8 cm x 8 cm) individuals could enter the middle compartment (33 cm x 22 cm), where the differently grained sandpapers were presented (see Fig. 2a). From the middle compartment, rats could enter the reward chambers, which were equal in size (16.5 cm x 19 cm) and positioned next to each other. Start and reward chambers could be manually closed by sliding doors. Each reward chamber contained a food bowl (Ø 10 cm, height: 5 cm). Differently coloured food bowls were chosen for visual distinction between the two chambers (white bowl in the right chamber, black bowl in the left chamber). Within each food bowl a Petri dish with holes in the lid was placed. Both Petri dishes contained a reward to exclude the possibility, that rats made their decisions only based on olfactory cues. Since the dishes were sealed, rats could not excess the rewards inside the petri dish. Rewards indicating the positive and negative condition were therefore presented on top of the Petri dish. As rewards we used honey loops (Honey Llama Loops, Kellogg Europe Trading, Dublin, Ireland).

Based on the study by Brydges and Hall^34^, differently grained waterproof sandpapers (klebemeister.eu, Adbeere Com Marketing Unternehmergesellschaft & Co. KG, Gerbrunn, Germany; 230 mm x 280 mm) served as tactile cues. In accordance with Brydges and Hall^34^, coarse sandpaper (type 60) and fine sandpaper (type 1200) were used to train the rats and served as reference cues for later analysis, whereas sandpaper of type 180 was used as the intermediate ambiguous cue during the test phase. In addition, we added two more ambiguous sandpapers (type 120 and type 400) during the test phase. The coarseness of the ambiguous cues was supposed to be in between the two learned reference conditions, with 180 being the middle condition and 120 and 400 being closer to the references (type 120 = near-coarse, type 400 = near-fine). It is important to note that the classification of the coarseness of sandpaper is not proportional, which is why the difference between type 400 and type 1200 seems more drastic than the difference between type 60 and type 120. We therefore based our reference cues and the middle cue on the paper by Brydges and Hall^34^ and chose the two additional sandpapers in a way that their coarseness was between the reference cue and the middle cue. Near-coarse and near-fine conditions were included to investigate whether the unknown sandpapers were actually rated in reference to the learned cues, which would be reflected in a graded response curve^19,35^. The ambiguous conditions are important to assess the cognitive judgement bias of the rats. If an individual chooses the reward chamber associated with the positive condition during an ambiguous trial, it is considered as optimistic because it anticipates a big reward. On the other hand, an individual’s response will be considered as pessimistic, if it enters the reward chamber of the learned negative condition (expectation of a small reward)(see Fig. 2b). During training and testing the sandpaper was secured to the floor of the middle compartment with magnets. In contrast to Brydges and Hall^34^, we introduced a large slider in addition to the sandpaper on the floor, that could divide the middle compartment in half. The slider was equipped with the same type of sandpaper presented on the floor and rats had to touch the sandpaper on the slider to open the way to the reward chambers. To avoid olfactory distraction caused by the smell of other rats, each individual had its own set of sandpapers. After each training or test the sandpaper sheets were cleaned under running water. The apparatus itself was wiped with ethanol before the first and in between sessions.

## Training phase

The training phase was divided in four different steps (Habituation, Pre-Training, Training I and Training II, see Table 1), which had to be successfully completed for the animal to be admitted to the test phase. During training, rats were habituated to the test apparatus, learned to associate the reference sandpapers (type 60 and type 1200) with big and small rewards and became accustomed to unrewarded trials. In the positive condition, individuals received half a honey loop when choosing the correct chamber, while in the negative condition only one-sixth of a honey loop was presented in the correct chamber. The incorrect chamber remained empty and rats were not punished for incorrect choices. A choice was defined as entering the reward chamber with all four paws and approaching the food bowl with the snout. Before training, each individual was assigned to a positive and negative condition, which consisted of the type of sandpaper, the reward chamber (left or right) and the reward size. This resulted in four different combinations for the positive conditions and the corresponding negative conditions. All combinations were counterbalanced across treatment groups and individuals (see Table S1 in the supplementary material). Individuals were also habituated to unrewarded trials (see Table 1) because the response to ambiguous sandpaper in the test phase was not rewarded. In general, the order of cues during training and testing was pseudo-randomised and care was taken that no sandpaper was presented more than three times in a row. Moreover, a training or test session was not allowed to start or end with an unrewarded trial. Between two unrewarded trials, rat had to experience both reference cues at least once. Training and test phase took place in the housing room under red light conditions between 9.30 and 16.00 and were conducted by the same experimenter. Each rat was trained once a day for a maximum time of 40 min and for five days a week, until it reached the corresponding learning criterion (see Table 1). At the beginning of each training or test phase, rats spent 1 min in the start box to acclimatise to the situation. The order, in which animals were tested, was randomised every day using the sample-function in Rstudio.

## Test phase

After the rats reached the final learning criterion (see Table 1), they were admitted to the test phase. The CJBT was conducted on three consecutive days to enable a sufficient presentation of ambiguous cues without over-presenting the ambiguous cues within a single session. The ambiguous sandpapers were classified as near-positive, middle or near-negative, depending on the learned conditions. Each ambiguous cue was presented twice per test day, leading to a total of 6 ambiguous trials and 12 trials each in the positive and negative condition. Ambiguous trials were not rewarded and care was taken that each ambiguous sandpaper followed both reference cues once to mitigate potential effects of the previously presented cue. Since environmental enrichment served as a treatment and a non-automated test procedure was used, which required the interaction of experimenter and rats, the experimenter was not blinded to the treatment or the rats’ responses (optimistic or pessimistic) to the ambiguous cues. However, due to the centred position of the experimenter to the apparatus, the randomisation of trials, the balanced combinations of sandpaper, reward chamber and reward size and the very fast reaction time of the rats, we consider the possibility of an influence by the experimenter to be very low.

**Table 1:** Summary of the different steps of the cognitive judgement bias test (CJBT). The training phase is divided in four steps (Habituation, Pre-training, training I and training II) followed by the test phase. Shown are the duration of each step as well as the number of trials, the aim and execution and the criteria to enter the next step.

Habituation				
Duration	Trials	Aim	Execution	Criteria for next step
1 day	At least 10 trials within 40 min	Habituation to the test apparatus and the rewards	No sandpaper No large slider Rats can explore the reward chambers and find equal rewards (a quarter Honey Loop) in both chambers	Each reward chamber must be entered at least five times and rewards need to be eaten

**Pre-Training**				
Duration	Trials	Aim	Execution	Criteria for next step
At least 1 day	30 only-correct within 40 min	Introduction of positive and negative condition Association of sandpaper, reward chamber and reward size	With sandpaper and large slider Negative condition: sixth of a Honey Loop Positive condition: half of a Honey Loop Incorrect chamber is closed, individuals can only enter the correct chamber	30 only-correct trials within 40 min If not: Repetition of pre-training until criterium is met

**Training I**				
Duration	Trials	Aim	Execution	Criteria for next step
At least 1 day	4 only-correct* 30 trials *with* correction trials within 40 min	Learning to choose the correct chamber ***	With sandpaper and large slider Both reward chambers are open In case of a wrong decision the trial is repeated (correction trial) After a maximum of two correction trials the incorrect chamber is closed to guide the rat in the correct chamber	80% correct choices (for both positive and negative condition) *and* < 10 correction trials ** If not: Repetition of Training I until criteria are met If less than 80% are correct *and* > 19 correction trials are needed, the rat returns to Pre-training

**Training II**				
Duration	Trials	Aim	Execution	Criteria for next step
At least 1 day	4 only-correct* 30 trials *without* correction trials within 40 min	Habituation to unrewarded trials	With sandpaper and large slider Both reward chambers are open 6 unrewarded trials: 3 positive trials 3 negative trials	80% correct choices (for both positive and negative condition) If not: Rat returns to Training I

**Test**				
Duration	Trials	Aim	Execution	Criteria for next step
3 consecutive days	4 only-correct* 30 trials *without* correction trials within 40 min	Rats have to respond to unknown and ambiguous cues Optimistic or pessimistic choices	With sandpaper and large slider Both reward chambers are open 6 ambiguous trials (unrewarded): 2 near-positive 2 middle 2 near-negative	Once an individual reaches the test phase it participates in the CJBT on all 3 days

* Each training or test day started with 4 only-correct trials (2 positive, 2 negative) to remind the rats of the correct association between sandpaper and reward chamber. During only-correct trials the incorrect chamber was closed and were therefore not part of the later analysis. ** In some cases, Training I was continued, although the criteria were accomplished. This was due to a break in training over weekends. Since the CJBT was conducted following Training II and lasted for three consecutive days, some individuals remained in Training I until the time schedule allowed for consecutive training and testing over four days. *** A choice was made as soon as the rat entered the reward chamber with all four paws and approached the food bowl with its snout.

## Sample size and statistical analysis

All rats were re-used from a previously conducted study, that investigated the effect of environmental novelty on paw preference in rats^33^. The regular enrichment change was already part of that study and was continued after the study was completed. The selection of rats for the current study was based on their previous experience: During their study, Groneberg et al.^33^ divided the rats in three groups. One group was housed under consistent enrichment (control group), whereas the other two groups experienced enrichment change of different durations. The enrichment regimes for the two treatment groups of the current study should be as different as possible. Therefore, we re-used the rats from the control group and rats, which experienced enrichment change for the longest time. This led to a sample size of 24 rats, with 12 rats having experienced only consistent enrichment in the past and 12 rats having encountered regular enrichment change for several months. In previous studies, sample sizes of 12 to 24 rats were sufficient to detect group differences in optimism level in relation to different housing conditions^23,36^. However, due to health issues that were not related to any experimental procedures, 3 rats had to be euthanised before the start of our experimental phase leading to a total sample size of 21 individuals (consistent-enrichment group: *n* = 10, enrichment-change group: *n* = 11). For euthanasia, carbon dioxide (CO_2_) was used, which was gradually induced in a 51.62 L chamber with a flow rate of 9 L per minute. Of the 21 rats, all individuals successfully completed the training and were admitted to the cognitive judgement bias test and included in the statistical analysis. A post hoc power analysis revealed that a high statistical power (> 0.9) could be achieved.

Data were analysed with R Statistical Software (version 4.5.0; R Core Team, 2025) and RStudio (version 2025.5.0.496) with packages lme4^37^, lmerTest^38^ and emmeans^39^. Graphs were created using the package ggplot2^40^. A generalized linear mixed model (GLMM) was fitted to examine the effect of treatment (consistent-enrichment or enrichment-change), condition (positive, near-positive, middle, near-negative, negative) and their interaction on the rats’ response (optimistic or pessimistic) to the different cues. The response was modelled as proportion data (proportion of optimistic responses). The numbers of presentations per cue type (reference cues = 36, ambiguous cues = 6) were used as weights within the model and the individuals’ ID was included as a random factor. Residuals were graphically checked for normality and homogeneity by using the DHARMa package^41^. After applying a type III ANOVA to the model (originating from the car-package^42^) to investigate main and interaction effects, we used the emmeans function for pairwise post hoc comparison with Bonferroni-Holm correction for multiple testing. Semi-partial (part) R² was assess as a measure of the effect sizes. It describes how much variance was explained by each predictor and their interaction within the model. Part R² was calculated using the package partR2^43^. Differences were considered significant when *p* ≤ 0.05. Differences with 0.05 < *p* < 0.1 were considered a trend.

For illustration of the results, we calculated an optimism score for each individual and each condition. The optimism score was calculated by dividing the difference of optimistic and pessimistic responses by the sum of optimistic and pessimistic responses (Optimism score = (optimistic – pessimistic )/ (optimistic + pessimistic)). Therefore, scores close to 1 indicate a high optimism level, whereas scores close to -1 represent a very low optimism level. The score is closely related to the proportion of optimistic responses used in the model, but improves readability of the results.

### Ethical note

All procedures were in accordance with the regulations covering animal experimentation within Germany (Animal Welfare Act) and EU (European Communities Council DIRECTIVE 2010/63/EU) and were approved by local (Amt für Gesundheit, Veterinär- und Lebensmittelangelegenheiten, Münster, Nordrhein-Westfalen) and federal authorities (Landesamt für Natur, Umwelt und Verbraucherschutz Nordrhein-Westfalen ‘LAVE (ehemals LANUV) NRW’, reference number 81-02.04.2021.A103). This study is reported in accordance with ARRIVE guidelines.

## Results

All rats required between 7 and 12 days of training (Ø 10.3 days), including habituation, pre-training, training I and training II, before reaching the criterion for being tested. The habituation took only 1 day. For pre-training, 1–3 days were needed (Ø 1.3 days). The first training step had an average duration of 7 days (minimum 4 days, maximum 9 days), and the second training step lasted 1 day with exception of one rat, which had to repeat this training step before reaching the test criterion. However, it should be mentioned again that some rats remained in training 1 even though they had already met the criteria for training 2 (see Table 1).

**Fig. 3:**
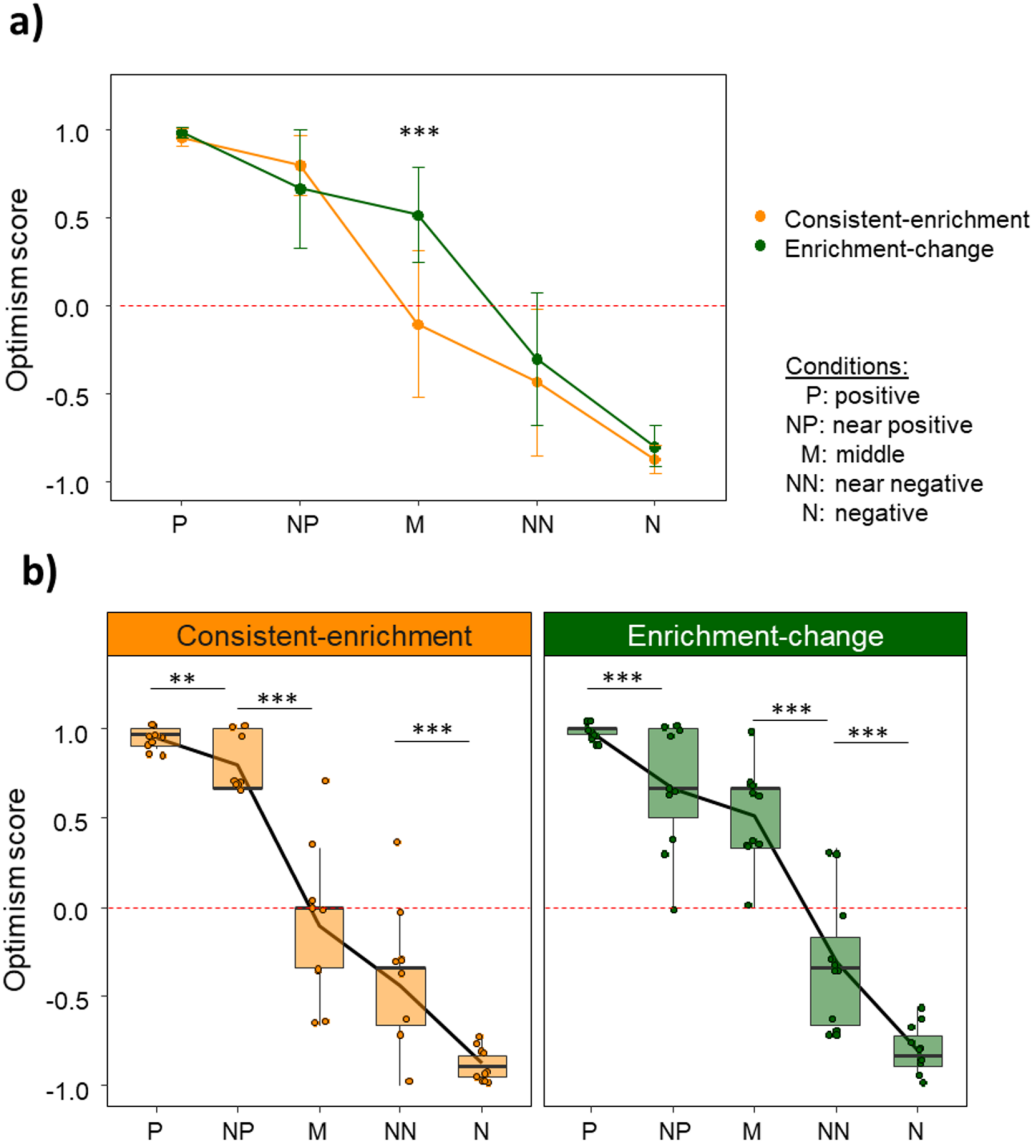
Comparison of optimism scores between treatment groups (**a**) and among conditions (**b**). The optimism score ranges from − 1 (least optimistic) to + 1 (most optimistic). The dashed line highlights an optimism score of 0. (**a**) The enrichment-change group had significantly higher optimism scores in the middle condition compared to the consistent-enrichment group. Optimism scores of both treatment groups are presented as means ± standard deviation for all five conditions, resulting in two grades response curves. (**b**) Significant differences among condition were found in both treatment groups. The graded response curves are presented and supplemented by data points representing the optimism scores of the single individuals. Additionally, the median and the first and third quartile are shown. Highlighted are the significant differences between adjacent conditions (see Table 3 for all post hoc comparisons). Sample size: *n* = 10 for the consistent-enrichment group and *n* = 11 for the enrichment-change group. Asterisks depict statistically significant differences: * *P* ≤ 0.05, ** *P* < 0.01, *** *P* < 0.001 (GLMM and post hoc comparison, Bonferroni-Holm corrected).

The optimism level was significantly affected by the treatment (GLMM: χ² = 11.65, df = 1, *p* < 0.001; part R² = 0.009, CI = [0, 0.121]), condition (GLMM: χ² = 285.32, df = 4, *p* < 0.001; part R² = 0.557, CI = [0.516, 0.603]) and their interaction (GLMM: χ² = 9.69, df = 4, *p* = 0.046; part R² = 0.025, CI = [0, 0.202]). Post hoc comparisons revealed a significant difference between consistent-enrichment and enrichment-change group for the middle condition (M) (Fig. 3a), with individuals of the enrichment-change group having a higher proportion of optimistic responses (estimate = -1.348 ± 0.395, z = − 3.413, *p* < 0.001; see Table 2 for the other post hoc comparisons). Apart from that, both treatments showed graded response curves with a decrease in optimism scores from positive to negative condition (Fig. 3b, for individual response curves see Figure S1 in the supplementary material). Post hoc comparisons between the different conditions revealed significant differences among all conditions except for the middle (M) and near-negative (NN) condition within the consistent- enrichment group and middle (M) and near-positive (NP) condition within the enrichment-change group (see Table 3 for all post hoc comparisons).

**Table 2:** Summary of pairwise post hoc comparisons of the optimism scores between the two treatment groups (consistent-enrichment and enrichment-change) for all five conditions (positive, near-positive, middle, near-negative, negative) *P*-values were corrected for multiple testing using the Bonferroni-Holm method. *P*-values in bold depict statistical significance (*p* ≤ 0.05).

Condition	Estimate	z-ratio	*p*-value
**Positive (P)**	-1.092 ± 0.685	-1.594	0.111
**Near-positive (NP)**	0.589 ± 0.548	1.706	0.282
**Middle (M)**	-1.348 ± 0.395	-3.413	**< 0.001**
**Near-negative (NN)**	-0.304 ± 0.393	-0.773	0.440
**Negative (N)**	-0.499 ± 0.282	-1.771	0.077

**Table 3:** Summary of pairwise post hoc comparisons of the optimism scores among conditions (positive, near-positive, middle, near-negative, negative) for both treatment groups (consistent-enrichment and enrichment-change). *P*-values were corrected for multiple testing using the Bonferroni-Holm method. *P*-values in bold depict statistical significance (*p* ≤ 0.05).

Consistent-enrichment group			
Comparison	Estimate	z-ratio	*p*-value
**P – NP**	-1.589 ± 0.560	-2.837	**0.009**
**NP – M**	-2.410 ± 0.504	-4.781	**< 0.001**
**M – NN**	-0.732 ± 0.388	-1.887	0.059
**NN – N**	-1.762 ± 0.359	-4.904	**< 0.001**
**P – M**	-3.999 ± 0.443	-9.019	**< 0.001**
**P – NN**	-4.731 ± 0.460	-10.275	**< 0.001**
**P – N**	-6.493 ± 0.421	-15.431	**< 0.001**
**NP – NN**	-3.142 ± 0.519	-6.053	**< 0.001**
**NP – N**	-4.904 ± 0.484	-10.130	**< 0.001**
**M – N**	2.494 ± 0.339	7.363	**< 0.001**

**Enrichment-change group**			
**Comparison**	**Estimate**	**z-ratio**	***p*** **-value**
**P – NP**	-3.27 ± 0.668	-4.898	**< 0.001**
**NP – M**	-0.475 ± 0.439	-1.076	0.282
**M – NN**	1.776 ± 0.388	4.577	**< 0.001**
**NN – N**	-1.567 ± 0.309	-5.079	**< 0.001**
**P – M**	-3.742 ± 0.647	-5.78	**< 0.001**
**P – NN**	-5.518 ± 0.636	-8.676	**< 0.001**
**P – N**	-7.085 ± 0.605	-11.704	**< 0.001**
**NP – NN**	-2.248 ± 0.421	-5.337	**< 0.001**
**NP – N**	-3.815 ± 0.373	-10.229	**< 0.001**
**M – N**	3.343 ± 0.335	9.982	**< 0.001**

## Discussion

In this study, we conducted a cognitive judgement bias test to examine the impact of different enrichment regimes on the emotional state of laboratory rats. Therefore, individuals were divided in two groups, in which enrichment items either remained the same or were recombined every week over a period of several months. The comparison between the treatment groups revealed that rats from the enrichment-change group responded more optimistically in an ambiguous situation than rats that experienced consistent enrichment.

First of all, the optimism scores of both treatment groups resulted in a graded response curve, which shows a monotonous decrease from positive to negative condition. This finding is in line with previous studies in rodents and other species^19^, 44]–^49^. It indicates that the rats likely interpreted the ambiguous cues in reference to the learned cues, which is a basic requirement for the validity of the test^19^. More interesting, however, is the significant difference in optimism scores between both groups for the middle condition of the CJBT. The middle cue represents, by definition, the highest degree of ambiguity and is therefore best suited to uncover potential differences in the optimism level^17,50^. In this study, rats that experienced a recombination of enrichment items twice a week over a period of several months, had significantly higher optimism scores in the CJBT than rats living with consistent enrichment. This clear difference in the optimism level between the two enrichment regimes is somewhat remarkable, since both housing environments include various enrichment items and should both be considered as highly enriched. In general, providing environmental enrichment is known to positively affect and improve welfare of laboratory rodents^51^, and previous CJBT studies could also show that provision of enrichment induced a positive cognitive bias in different species^16^. Nevertheless, a few studies did not report any effects of enrichment provision and changed housing conditions on the animals’ optimism levels^27,28,52^. Although the provision of enrichment was shown to improve welfare-related parameters in rats^51,53^, regular change of enrichment items was rarely studied so far. Moreover, results obtained from studies in mice were inconclusive. A study by Gross et al.^54^, for example, found no effect on welfare-related parameters following regular enrichment change, whereas Ramírez-Rodríguez et al.^55^ found reduced anxiety-like behaviour in mice in response to changing enrichment conditions. With our study, we were able to provide further evidence that a regular change of enrichment can positively affect laboratory rodents. However, it should be noted that only female rats were used for the current study. A review article by Lagisz et al. (2020) indicated that cognitive judgement bias tests might be influenced by sex, due to sex differences in neurobiology or learning abilities^50^. Moreover, possible sex-specific influences of the treatment should be taken into account, since environmental enrichment can sometimes have different effects on male and female rodents^8,56^. Further research on male rats is therefore needed in order to draw clear conclusions about the effect of enrichment change on male rats.

Another aspect, that was previously discussed in the cognitive judgment bias literature, is the question whether novelty can be a confounding factor for ambiguous cue interpretation^57^. In a spatial CJBT, for example, the response to the novel and ambiguous cue is closely related to an individual’s exploratory behaviour, which could also lead to masking or misinterpretation of group differences^58^. Moreover, it was found that rats in an unpredictable environment responded more optimistically than rats from a predictable environment, although unpredictability was assumed to elicit a negative affective state. This was explained by a higher sensitivity to novelty by individuals from the predictable housing condition because they were not used to change^59^. With regard to our study, one could assume that individuals from the consistent-enrichment group would react more sensitive to novelty in form of the presentation of new sandpapers, since they were not used to changes in their environment. However, we could observe that rats of both groups were very fast and not hesitant in making decisions during the test phase. Therefore, we do not assume that the rats of the consistent-enrichment group were negatively affected by experiencing a new type of sandpaper during ambiguous trials.

To explain the difference in optimism level between the groups, it is important to note that our rats experienced these enrichment regimes for a total time of more than 6 months. This stands in contrast to many other studies that conducted cognitive judgement bias tests directly after changing the housing conditions, e.g., after transferring animals from barren to enriched housing^23,25,60^. Therefore, it seems plausible that in the current study, rats from the consistent-enrichment group habituated to their enrichment over time. Such habituation effects could already be shown in other contexts in the past, reflected by a reduced interaction with enrichment items for example^61–63^. As a consequence, also highly enriched cages might become monotonous and less stimulating over time. In contrast, by frequently changing the enrichment items within a cage, the stimulating value of the enrichment could be maintained. Moreover, a frequent change of enrichment is also reasonable from an ecological perspective, as animals are rarely confronted with stable and never changing environmental conditions in their natural habitats^29,30^. It is therefore worth considering to use frequent enrichment changes as a refinement method for laboratory rats. However, one must be aware that not only the enrichment items used in the current study, but also the cage size did not conform to the standard housing conditions in most laboratories. It might be possible that a comparison with a frequently used standard housing condition (type IV cage for example), might reveal a more optimistic cognitive bias in the consistent-enrichment group. Nevertheless, a regular enrichment change could possibly also be incorporated in standard cages, such as varying shelter or nesting materials, to improve rats’ welfare. But to confirm this assumption, further research is needed. Regarding the practicability of a regular enrichment change, it should be emphasised that the enrichment change in the current study was based on the recombination of already familiar enrichment items. Considering the fact that the implementation of refinement strategies can entail organisational obstacles^64^, the recombination of already familiar objects represents a decisive advantage for the practicability of the enrichment change. It suggests that it is not necessary to continually purchase and provide novel enrichment items because the recombination of existing enrichment can already be efficient to positively affect the animals’ welfare.

In conclusion, our study suggests that regular enrichment change could be a promising refinement method for laboratory rats. Recombining familiar enrichment items can be a simple but effective way to positively impact their welfare. Further research would be useful to translate our finding to other laboratory species and investigate the influence of this method on a broader spectrum of welfare-related parameters.

## Supplementary Information

Below is the link to the electronic supplementary material.

Supplementary Material 1

## Data Availability

Data and R code for analysis are publicly accessibly in an online repository (https://doi.org/10.5281/zenodo.17115895). For further requests, please contact Elena Groneberg.
