# Supplementary material for: The influence of regularly changing enrichment on the cognitive judgement bias of laboratory rats
Source: Sci Rep. 2025 Oct 8;15:35049. doi: 10.1038/s41598-025-22088-x (PMC12508032; doi:10.1038/s41598-025-22088-x)
Supplement: Supplementary file 1 — Supplementary Material 1 [file 41598_2025_22088_MOESM1_ESM.pdf]

Supplementary material

Detailed description of enrichment items

In category shelters, we used a wooden shelter (willow bridge, beeztees, Netherlands; ca. 28 cm x 17 cm), a shelter made of cardboard (rat house, ca. 21 cm x 21 cm x 10 cm) and a shelter made of red semi-transparent plastic (guinea pig house, 20.5 cm x 15.7 cm x11.5 cm), both purchased from ZOONLAB GmbH Animal Husbandry Experts, Germany. As tunnels we used a cuboid tunnel made of ceramic (ceramic tunnel, TRIXIE Heimtierbedarf GmbH & Co. KG; 19.5 cm x 9 cm x 9 cm), a cuboid tunnel made of red semi-transparent plastic (15 cm x 9 cm x 9.5 cm) and a cylindric tunnel made of cardboard (length: 12.5 cm, Ø 9 cm), both purchased from ZOONLAB GmbH Animal Husbandry Experts, Germany. The category hanging enrichment consisted of items, that hang from the top of the cage and could be used for climbing or resting. Cages were either enriched with a tea towel, which was used as a hammock (50 x 70 cm), a wooden swing (rody swing, Karlie GmbH, Germany, 20 cm x 20 cm) or two flexible rope perches for climbing (rope perch, TRIXIE Heimtierbedarf GmbH & Co. KG; 37 cm, Ø 16 mm and 66 cm, Ø 18 mm). The last category gnawing enrichment included three items of different materials like a willow ball (Ø 7cm), a gnawing root (gnawing root, Bunny Tierernährung GmbH, Germany) and a cardboard toy (toy carrot, Flamingo Pet Products, Belgium; 3.5 cm x 9 cm x 3.5 cm).

Supplementary file for "Embrace the change: How regularly changing enrichment impacts the cognitive judgement bias of laboratory rats." – Groneberg E, Hoppe LN, Richter SH, Kaiser S

Table S1: **Overview of sandpaper and reward chamber used as positive condition for each individual**. The presentation of the big reward (positive condition) was balanced across both reward chambers (left and right) and both types of sandpaper (1200 = fine, 60 = coarse). All individuals were allocated to their combination of sandpaper and reward chamber before the training started.

ID	Treatment group	Positive condition	Reward chamber
57	Enrichment-change	1200	Right
58	Enrichment-change	1200	Left
59	Enrichment-change	60	Right
60	Enrichment-change	60	Left
65	Consistent-enrichment	60	Right
66	Consistent-enrichment	60	Left
68	Consistent-enrichment	1200	Right
70	Enrichment-change	1200	Right
71	Enrichment-change	1200	Left
72	Enrichment-change	60	Left
77	Consistent-enrichment	60	Right
78	Consistent-enrichment	60	Left
79	Consistent-enrichment	1200	Right
80	Consistent-enrichment	1200	Left
81	Enrichment-change	1200	Right
82	Enrichment-change	1200	Left
83	Enrichment-change	60	Right
84	Enrichment-change	60	Left
89	Consistent-enrichment	60	Right
90	Consistent-enrichment	60	Left
92	Consistent-enrichment	1200	Left

Supplementary file for “Embrace the change: How regularly changing enrichment impacts the cognitive judgement bias of laboratory rats.” – Groneberg E, Hoppe LN, Richter SH, Kaiser S

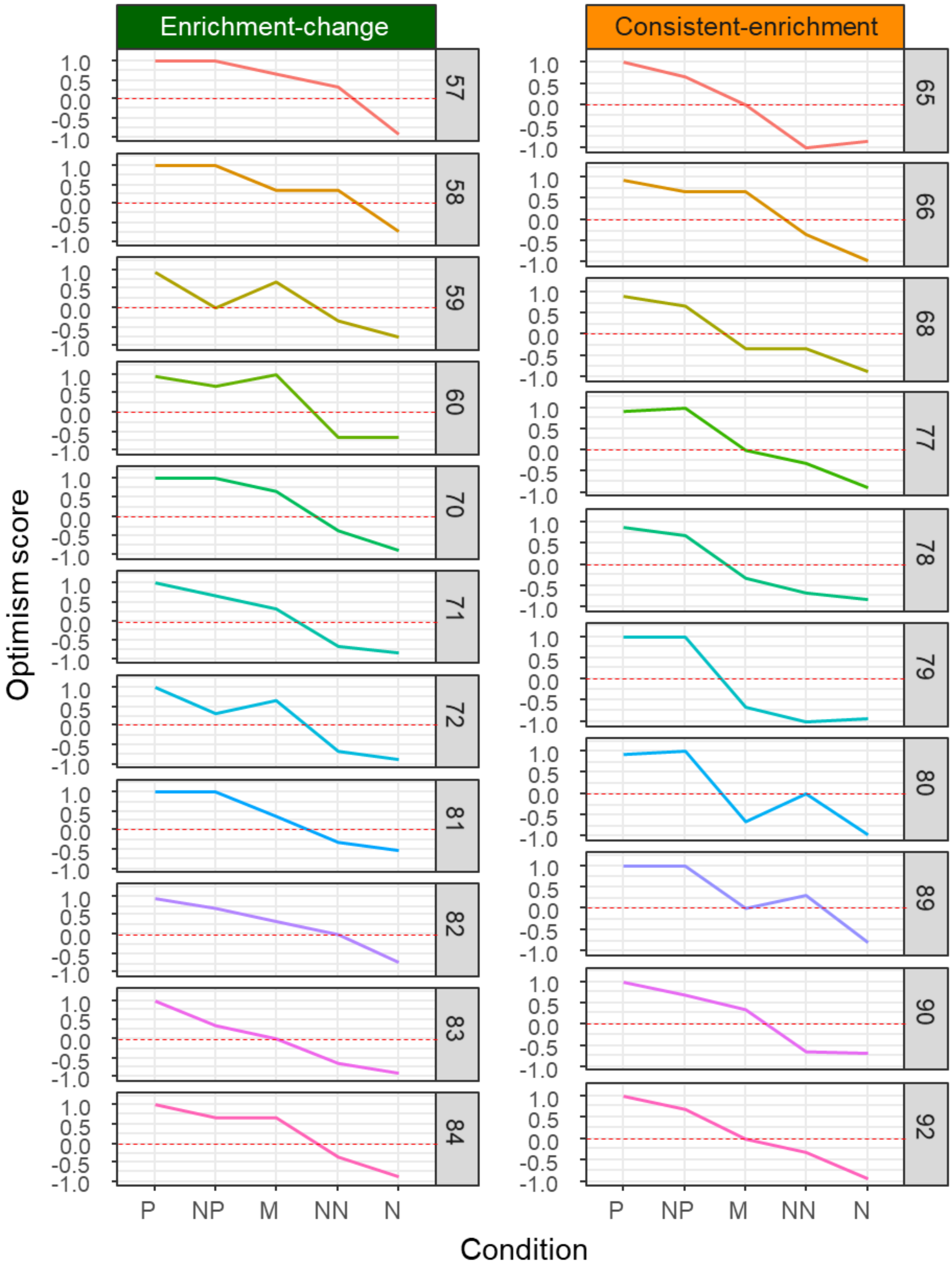

Figure S1: Individual graded response curves of all rats from both treatment groups. The optimism score ranges from -1 (least optimistic) to +1 (most optimistic). The dashed line highlights an optimism score of 0.
